# Enhancing the Photoelectrochemical Performance of a Nanoporous Silicon Photocathode through Electroless Nickel Deposition

**DOI:** 10.3390/nano13182552

**Published:** 2023-09-13

**Authors:** Yao-Hung Yeh, Chiao-Li Chang, Zi-Chun Tseng, Vincent K. S. Hsiao, Chun-Ying Huang

**Affiliations:** Department of Applied Materials and Optoelectronic Engineering, National Chi Nan University, Puli 54561, Taiwan; s104328508@mail1.ncnu.edu.tw (Y.-H.Y.); s109328050@mail1.ncnu.edu.tw (C.-L.C.); s109328007@ncnu.edu.tw (Z.-C.T.)

**Keywords:** photoelectrochemical, nanoporous silicon, nickel, water-splitting catalysts

## Abstract

Renewable energy sources, particularly solar energy, are key to our efforts to decarbonize. This study investigates the photoelectrochemical (PEC) behavior of nanoporous silicon (NPSi) and its Ni-coated hybrid system. The methods involve the application of a Ni coating to NPSi, a process aimed at augmenting catalytic activity, light absorption, and carrier transport. Scanning electron microscopy was used to analyze the morphological changes on NPSi surfaces due to the Ni coating. Results demonstrate that the Ni coating creates unique structures on NPSi surfaces, with peak PEC performance observed at 15 min of coating time and 60 °C. These conditions were found to promote electron-hole pair separation and uniform Ni coverage. A continuous 50-min white light illumination experiment confirmed stable PEC fluctuations, showing the interplay of NPSi’s characteristics and Ni’s catalytic effect. This study provides practical guidance for the design of efficient water-splitting catalysts, contributing to the broader field of renewable energy conversion.

## 1. Introduction

The quest for sustainable development and the reduction of carbon emissions has led to the development of renewable energy sources such as solar energy, wind energy, geothermal energy, hydroelectric power, and biomass energy [1]. However, economic viability remains a challenge for many emerging energy technologies. Some renewable sources, such as biomass energy and geothermal energy, have environmental constraints that limit their applicability across different regions [2]. Conversely, solar energy is abundant and universally available. Researchers have demonstrated that solar energy can satisfy global energy demands for an entire year, underscoring its reliability [3,4]. Two types of systems are mainly used to convert solar energy into electricity. The first system involves photovoltaic solar cells, where the photovoltaic effect is used to convert light energy into electrical energy. The second system, the photocatalytic hydrogen production system, comes in two subtypes that are distinguished by their principal mechanisms: photocatalytic reaction and the photoelectrochemical (PEC) process [5,6]. The development of both types of photocatalytic hydrogen production systems relies on the development of novel semiconductor photocatalytic materials [7,8]. When semiconductor materials are exposed to light with energy that exceeds the electron energy gap of the material, electrons can be excited from the valence band to the conduction band. This process generates electron-hole pairs that are transferred to the surface of the material, resulting in electrochemical reactions to produce hydrogen [9]. Therefore, the selected semiconductor materials must possess an energy band distribution that corresponds with the redox potential of water [10,11,12].

The working principle underlying the use of photocatalysis to decompose water and produce hydrogen gas through PEC reactions involves the excitation of the surfaces of semiconductor materials by photon energy to generate electrons and holes, leading to charge separation [9]. Subsequent redox reactions on the surface of the semiconductor material decompose water into hydrogen and oxygen. In this process, the bandgap size of the semiconductor material becomes a crucial factor affecting the efficiency of photocatalysis. A smaller bandgap results in a material surface that is better able to absorb light, which, in turn, results in greater photoelectric conversion efficiency [7]. In addition to bandgap size, other characteristics of semiconductor materials, such as crystal structure, surface morphology, and charge transfer rate, contribute to the efficiency of photocatalysis [13]. Strategies for improving photoelectric conversion efficiency include the preparation of semiconductor structures of different shapes and sizes. These structures can increase the material’s surface area enhancing light absorption rate and charge separation efficiency [14]. Moreover, photocatalytic efficiency can also be improved by modifying the semiconductor material’s surface using metals, semiconductors, and organic molecules [9].

The principle of PEC hydrogen production relies on the photoelectric effect in semiconductor materials [14]. When the energy of incident light equals or exceeds the bandgap of the semiconductor, the light energy is absorbed, causing valence band electrons to jump to the conduction band, generating photogenerated electrons and holes. These particles migrate to the surface of the semiconductor, where they undergo redox reactions with water at the anodic contact surface, producing oxygen and hydrogen. In an ideal state, the application of a bias voltage of 1.23 V or more would enable hydrogen and oxygen production through water electrolysis using electrochemistry. However, this process requires substantial energy and is not cost effective. Therefore, scientists aim to use solar energy to drive PEC reactions for water decomposition and hydrogen production. The selection of materials for PEC water splitting must account for factors such as material bandgap, photogenerated charge transfer, and stability in the electrolyte to achieve optimal hydrogen production efficiency [15].

When a semiconductor material interfaces with a solution containing redox species in a PEC hydrogen production system, the Fermi level of the semiconductor strives toward equilibrium with the chemical potential of the solution [16]. In the case of a p-type semiconductor, where the Fermi level is lower than the chemical potential of the solution, electrons migrate towards the semiconductor, forming a space charge layer and generating an electric field at the semiconductor surface [17]. Upon exposure to light with energy greater than the semiconductor bandgap, electron-hole pairs are generated. Using a p-type semiconductor as the working electrode leads to reduction reactions at its surface, which produces hydrogen gas. Conversely, using a platinum electrode as the counter electrode leads to oxidation reactions occurring at its surface, which produces oxygen gas. Through this process, light energy enables the decomposition of water into hydrogen and oxygen [18].

This study explores the utilization of p-type nanoporous silicon (NPSi) as a photocathode, with a particular focus on enhancing photocatalytic performance through the deposition of Ni metal onto the island-shaped surface of NPSi using a straightforward electroless deposition process. A distinctive aspect of our work is the utilization of the island-shaped surface morphology of NPSi. This innovative approach significantly increases the reaction surface area, improving photocatalytic performance. This unique methodology sets our study apart from conventional techniques. Our research uncovers the intricate interplay between catalytic activity, enhanced light absorption, and the modulation of charge carrier dynamics, all of which result from the Ni coating on NPSi. This synergy represents a novel contribution to the advancement of photoelectrochemical (PEC) technology. We systematically investigate various Ni deposition times and temperatures, establishing optimal conditions for enhancing PEC efficiency. This approach offers a fresh perspective on the relationship between material morphology and optoelectronic properties. Importantly, we observe a consistent photocurrent density throughout 50 min of continuous white light exposure, underscoring the effectiveness of the Ni−NPSi hybrid system in maintaining efficient PEC performance. This has potential implications for the utilization of solar fuel. The island-shaped surface morphology of NPSi increases the reaction surface area and provides improved photocatalytic performance. NPSi is formed by immersing a p-type single crystalline Si semiconductor material in a hydrofluoric acid (HF)-based etching solution; NPSi is used as the anode, and a platinum sheet is used as the cathode. By changing the current density, Si materials with different nanostructures are etched out. This study also describes the process of PEC measurement, assessing the photocurrent as a function of applied bias voltage. The application of Ni coating onto the surface of NPSi has considerable potential as a means to enhance PEC conversion efficiency. This enhancement results from the interplay of catalytic activity, light absorption enhancement, and modulation of charge carrier dynamics, underscoring its role in advancing PEC technology. Scanning electron microscopy (SEM) analysis was used to determine changes in surface morphology caused by Ni deposition and their effects on PEC efficiency, reflectance behavior, and photoluminescence (PL). The intricate relationship between these modifications and functional outcomes underscores the complex nature of these phenomena. Tests under various Ni deposition times reveal the synergy between surface morphology, charge dynamics, and catalytic activity. The prominence of the 15-min deposition duration in attaining optimal PEC efficiency suggests a balance between catalytic site coverage and charge transport pathways. The correlation between Ni deposition durations, surface morphology, and PEC performance highlights the relationship between material morphology and optoelectronic properties. Surface protrusions observed following a 15-min Ni deposition enhanced light absorption and charge separation efficiency, enhancing PEC performance. The relationship between Ni deposition temperature, material morphology, and PEC performance underscores the role of temperature in the optimization of optoelectronic efficiency. Enhanced PEC performance at a 60 °C deposition temperature is attributed to improved Ni-adhesion, electronic structure, and balanced Ni cluster distribution. Stable photocurrent density, oscillating between −4 and −5 mA/cm² during 50 min of continuous white light exposure, underscores the equilibrium between photon-induced charge generation, catalytic activity, and voltage-induced drift. This finding highlights the effectiveness of the Ni−NPSi hybrid system in maintaining efficient PEC performance, with potential implications for solar fuel use.

## 2. Materials and Methods

**Figure 1:**
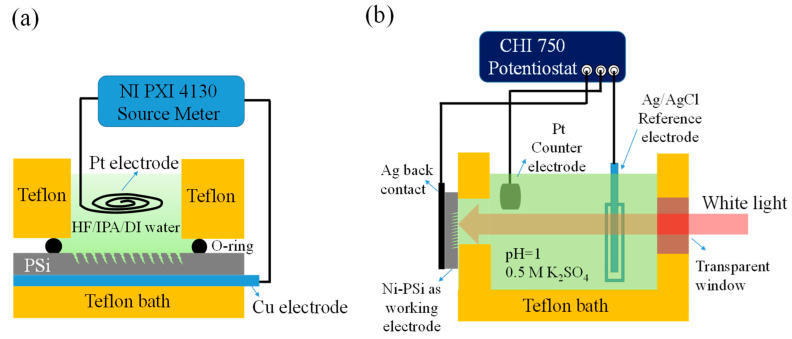
Schematic of (**a**) electrochemical etching setup used to fabricate NPSi and (**b**) PEC measurement setup with Pt as the counter electrode, NPSi as the working electrode, and Ag/AgCl as the reference electrode.

Before etching, the p-type single crystalline Si wafer was cut into pieces measuring 1.6 cm × 1.6 cm using a cutting machine. The surface of the Si was cleaned to eliminate organic and inorganic contaminants using deionized water (DI water), ethanol, and acetone and subsequently dried using a nitrogen gun. For electrochemical etching, the Si sample was secured in a vertical Teflon container, using a platinum sheet as the negative electrode and a 0.1-mm-thick platinum sheet as the electrode. The power supply used was an NI PXl-4130, as shown in Figure 1a. Subsequently, an electrochemical etching solution with a fixed concentration (HF: isopropanol: DI water) of 1:2:1 was added [19], and the etching conditions were adjusted according to specific current density and time. After etching for the prescribed duration, the sample was removed, and the excess etching solution was cleared using anhydrous alcohol. The NPSi was then rinsed with DI water for 1–2 min and dried with nitrogen gas. Isopropanol was added to the etchant to prevent the formation of smaller hydrogen bubbles that might hinder the etching process, and DI water was added to dilute the concentration of the HF solution (thereby preventing overetching).

The spontaneous deposition method was used to deposit Ni metal onto the NPSi surface. After electrochemical etching, the NPSi was positioned in a vertical Teflon container, and a nonelectrolytic Ni plating solution was prepared by combining NiSO_4_·6H_2_O as a Ni source, (NH_4_)_2_SO_4_ as a reducing agent, and (NH_4_)F as a stabilizing agent [20]. Using a micropipette, we transferred the solutions to a beaker and heated them on a heating plate. The temperature was monitored with a thermometer, and time was tracked with a timer. The bath temperature was maintained at different temperatures for different deposition durations. The test sample was then removed, thoroughly rinsed with DI water, and dried using a nitrogen gun.

Following the Ni deposition, conductive silver paste was applied to the back of the NPSi sample. This application required meticulous attention to prevent any leakage current that could affect the PEC properties of the measurement. In the first step of the process, the sample was laid flat, and the silver paste was applied to the back of the NPSi sample, ensuring coverage. During this application, care was taken to prevent leakage current between the contact metal and the sample. The importance of an even and controlled application of the silver paste was emphasized. The silver paste was then left to dry at room temperature, finalizing the working electrode, which was prepared for further measurements or experiments aimed at studying the PEC properties of the NPSi sample.

The PEC measurement setup used in this experiment is depicted in Figure 1b. A thin Ni film was deposited onto an NPSi sample, which had been created through electrochemical etching. The PEC behavior and hydrogen generation capacity of the sample were assessed under acidic conditions using a three-electrode electrochemical measurement system provided by CH Instruments. The electrolyte for the experiment was an acidic solution with a pH ranging from 1 to 1.2. It was prepared by combining 21.78 g of potassium sulfate (K_2_SO_4_) at a concentration of 0.5 M in a 500-mL beaker. Subsequently, 250 mL of water was added, followed by stirring with a magnetic stir bar placed on a hotplate stirrer until complete dissolution. The pH meter guided the gradual addition of sulfuric acid (H_2_SO_4_) until the pH value reached the desired range. During the PEC measurement, the sample surface was illuminated using 24 W of white light. The potential applied to the system was scanned from 0 to –1.2 V at a scan rate of 100 mV/s. The working electrode was connected to a reference electrode (Ag/AgCl) and an auxiliary electrode. A bias voltage was subsequently applied to enable the measurement process.

## 3. Results

### 3.1. PEC Performance, Reflectance, Photoluminescence (PL), and Surface Morphology of NPSi

**Figure 2:**
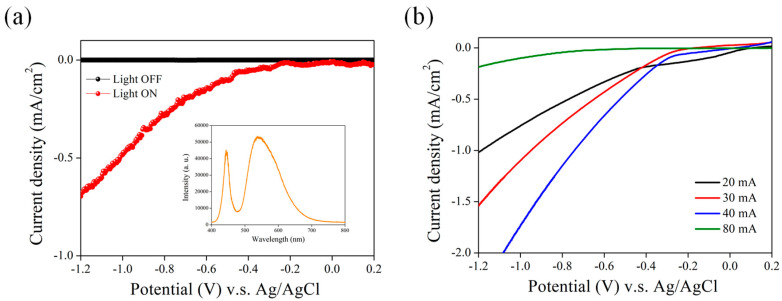
(**a**) PEC current density (J)–voltage (V) curves of NPSi photocathodes before (light OFF) and under white illumination (light ON). The J–V curves are scanned from −1.2 V vs. Ag/AgCl to 0.2 V in 0.5 M sulfuric acid solution. The inset shows the optical spectrum of the white light source. (**b**) PEC J-V curves of NPSi photocathodes etched using different currents.

Studies have demonstrated that Si materials with nanostructures perform well as photoelectrodes that enhance light response in PEC measurement [21]. For example, n-type Si nanostructures created through electrochemical etching have demonstrated improvements in photocurrent and quantum efficiency [22,23,24,25]. This enhancement is attributed to the reduction in surface reflectivity and the increase in resistance to light corrosion in acidic solutions caused by the nanostructures on the silicon surface. Conversely, p-type Si nanostructures have been found to be effective in both suppressing dark current and increasing PEC performance [26,27,28,29,30,31]. Figure 2a illustrates the PEC current density versus the potential of the working electrode NPSi before (light OFF) and after white light illumination (light ON). The optical spectrum of white light is shown in Figure 2a (inset). Using the Pt counter electrode as the anode and NPSi as the photocathode for PEC measurement, potentials were assessed against an Ag/AgCl reference electrode. When illuminated, NPSi can absorb photons to excite electrons, promoting them from the valence band to the conduction band, thereby generating photocurrent [32]. This transition highlights the role of light in enhancing photocurrent density. At a potential of −1.2 V relative to Ag/AgCl, the dark current density (light OFF state) of the NPSi photocathode was approximately −0.24 μA/cm^2^. Notably, under the same bias, the PEC current density of NPSi reached −0.68 mA/cm^2^ when illuminated by white light. This observation confirmed that the photocurrent density increase is linked to the surface properties of NPSi. Its nanoporous structure and surface properties enable NPSi to absorb light more effectively and convert it into a photocurrent [33]. This study further illustrates that adjustments to the pore structure and surface properties of NPSi can lead to more efficient light energy conversion. Previous studies have found that changing the electrochemical etching conditions alters the surface morphology of NPSi, thereby changing its PEC behavior [34]. By keeping the etching time constant and systematically varying the etching current, we were able to investigate the PEC behavior of NPSi samples fabricated under different etching currents, maintaining uniform light conditions. Figure 2b presents the variations in PEC current density under different electrochemical etching conditions. We observed the photocurrent density of NPSi after exposure to different current densities. When NPSi was subjected to illumination, the energy of the photons excited electrons within the material, causing them to transition from the valence band to the conduction band, thereby generating photocurrent [35]. Under a bias of −0.8 V, the electrochemical current density of NPSi reached −0.6, −0.75, −1.3, and −0.2 mA/cm^2^ observed in the NPSi etched using 20, 30, 40, and 80 mA currents, respectively. This outcome suggests that the increase in photocurrent density may be possibly influenced by the microporous structure and surface properties of NPSi [36]. These characteristics enable NPSi to absorb light more effectively and convert it into a photocurrent. This experiment corroborates the notion that more efficient light energy conversion can be attained by tuning the pore structure and surface properties of NPSi.

**Figure 3:**
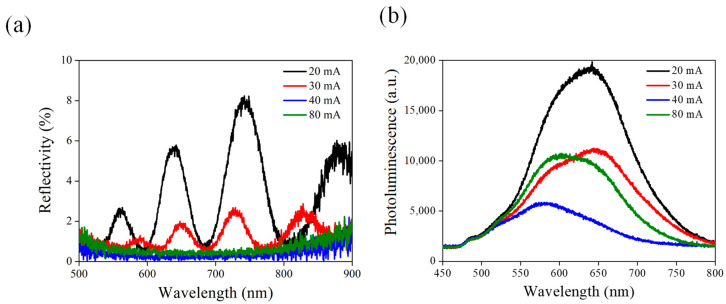
(**a**) Reflection and (**b**) PL spectra of NPSi etched using different currents.

Following the assessment of the PEC behavior of NPSi under various current etching conditions, further investigation was undertaken through reflectance testing to gain deeper insights into the effect of surface structures on the PEC performance of NPSi (Figure 3a). An analysis of the reflectance spectra of NPSi under different current etching conditions revealed a consistent reduction in reflectance as current density increased. This observation supports the previously posited connection between etching current and enhanced photocurrent in NPSi. Interestingly, at an etching current of 80 mA, the lowest reflectance was achieved, yet no marked improvement in PEC current occurred. A possible explanation may be related to the combined influence of the pore structure of NPSi on carrier transmission, offsetting the benefits of low reflectance. This factor might elucidate the modest increase in photocurrent despite the presence of low reflectance. Furthermore, a comprehensive analysis of the photoluminescence (PL) of NPSi was conducted under distinct current etching conditions (Figure 3b). This was done to explore the relationship between surface structures and the PEC performance of NPSi. As the etching current of NPSi increased, a discernible reduction in PL intensity was observed concurrently with a significant enhancement in PEC performance. This phenomenon may have been due to the complex interaction between the structural attributes and the band characteristics of NPSi. Particularly at higher etching current conditions, the pore structure of NPSi seemed to evolve into a more uniform and densely packed configuration. This change likely reduced the recombination rate between electrons and holes, thereby lessening the generation of PL. However, under an etching current of 80 mA, an unexpected increase in PL performance of NPSi was observed. This unusual trend could be linked to the presence of defect states in the surface pore structure, potentially aiding and amplifying the generation of PL.

**Figure 4:**
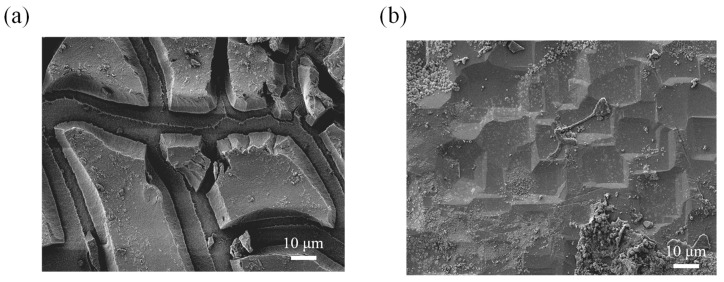
SEM image of NPSi etched using (**a**) 40 mA and (**b**) 80 mA current.

Further investigation into the surface morphology of NPSi was conducted using SEM, with the results depicted in Figure 4. Distinct island-like structures were observed on the surface of NPSi samples subjected to etching at 40 mA (Figure 4a). However, samples treated with an 80 mA etching current did not exhibit such pronounced island formations (Figure 4b). Strikingly, a correlation between the observed surface characteristics and the photocurrent density became evident, particularly under the 80 mA etching condition. The existence of island-like structures at 40 mA likely enhanced the surface electric field locally. This enhancement could promote the mobility and distribution of charge carriers, resulting in heightened photocurrent density. Conversely, the scarcity of conspicuous island-like structures at 80 mA might be ascribed to variations in surface defects, resulting in reduced PEC current density.

### 3.2. PEC Performance, Reflectance, Photoluminescence (PL), and Surface Morphology of Ni-Deposited NPSi

**Figure 5:**
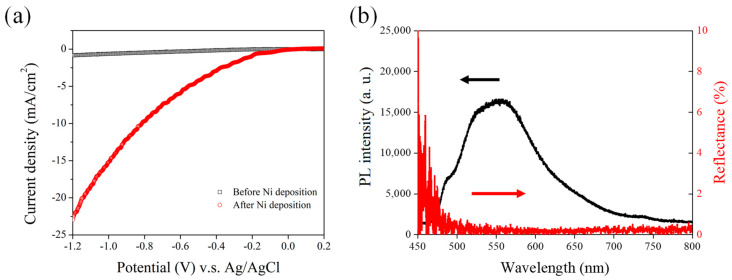
(**a**) PEC J–V curves of NPSi before and after Ni deposition under white illumination. The J–V curves are scanned from −1.2 V vs. Ag/AgCl to 0.2 V in 0.5 M sulfuric acid solution. (**b**) Reflection and PL spectrum of Ni-coated NPSi.

Previous studies on PEC have integrated submonolayer metal or metal oxide deposits onto the NPSi surface [37,38,39,40,41,42,43,44]. Notably, the application of Ni deposition on the NPSi surface leads to marked changes in its PEC behavior. Those studies have underscored the role of nanosized metal catalysts in improving the efficiency of PEC water splitting, especially under negative bias conditions [39]. Here, an analysis of the PEC properties of NPSi, both before and after Ni deposition, is illustrated in Figure 5a. It reveals that following Ni deposition, the PEC current density of NPSi experiences a substantial increase, with an enhancement of approximately 22.5 mA/cm^2^ under an applied bias of −1.2 V, closely resembles that of the TiO_2_-Si photocathode reported in prior research [45]. The factors contributing to the heightened PEC efficiency following deposition are multifaceted. The primary reason for improved PEC performance following Ni deposition may be attributed to the role of Ni as an effective catalyst, which promotes the progression of the PEC water-splitting reaction. Ni on the NPSi surface provides additional active sites, accelerating the generation of hydrogen and oxygen. As a catalyst, Ni lowers the energy barrier for hydroxide ion formation, reducing energy consumption during the PEC process and enhancing photoelectrochemical conversion efficiency. A secondary contribution to improved PEC performance comes from the increased light absorption capacity of NPSi following Ni deposition. The distinctive porous structure of NPSi enables the absorption of light across the visible and near-infrared spectra. Embedding Ni onto the NPSi surface further enhances photon absorption, increasing the efficiency of photon energy utilization and subsequently elevating PEC efficiency. Furthermore, Ni deposition aids in modulating the surface electronic structure of NPSi, thereby regulating the dynamics of charge carrier transport and recombination. This modulation suppresses carrier recombination, encouraging the production of photoelectrons and expediting charge carrier transport. Notably, Ni deposition also induces modifications in the band structure of NPSi, creating an interface conducive to efficient carrier separation and transport.

Following the deposition of Ni onto the surface of NPSi, both low reflectance and consistent fluorescence characteristics were preserved, as depicted in Figure 5b. This observation carries substantial implications for enhancing the efficiency of PEC processes. Upon examination, one can see that Ni deposition does not disrupt the low reflectance properties of the NPSi surface. Even with the modification in surface composition due to Ni incorporation, NPSi continues to exhibit reduced reflection across the incident light spectrum. This sustained low reflectance is instrumental in influencing photon absorption efficiency, a crucial factor in PEC systems [37]. By sustaining low reflectance, the Ni-deposited NPSi interface ensures a greater percentage of incident photons are absorbed, thus contributing to the enhanced generation of photoelectrons during PEC reactions. The consistency of PL following Ni deposition should be investigated. The PL intensity of NPSi remains stable despite the introduction of Ni to its surface. This stability highlights the intrinsic stability of the PL characteristics of NPSi when exposed to Ni. This consistency could be attributed to the coordinated interaction between the surface properties of NPSi and the nature of Ni deposition. Although Ni deposition introduces modifications, these alterations do not cause noticeable perturbations to the PL properties of NPSi. The importance of these observations is underscored when evaluating their effects on PEC performance. The continued low reflectance is vital for enhancing photon absorption and subsequent photoelectron generation, thereby enhancing the overall efficiency of PEC reactions. Moreover, the unaltered PL characteristics following Ni deposition indicate that the charge carrier dynamics responsible for PL remain largely unaffected. This consistency is advantageous for maintaining the stability and efficiency of charge separation and transport processes, both of which are integral to PEC performance. In summary, the simultaneous maintenance of low reflectance and consistent PL in Ni-deposited NPSi creates a favorable condition for advancing PEC technology. The insights drawn from this phenomenon open avenues for further exploration, potentially guiding novel approaches for optimizing photoelectrode materials in pursuit of highly efficient and stable PEC systems.

**Figure 6:**
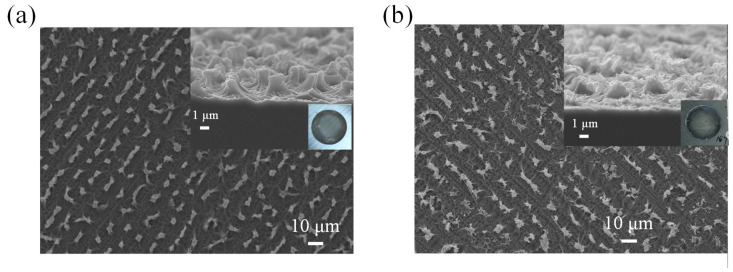
SEM image of NPSi (**a**) before and (**b**) after Ni deposition. Both insets illustrate the cross-sectional SEM images and photos of NPSi and Ni-coated NPSi samples.

The SEM analysis of NPSi before and after Ni deposition offers valuable insights into how changes in surface morphology affect PEC performance, reflectance properties, and fluorescence characteristics. Prior to Ni deposition, the SEM images revealed a distinct surface topography characterized by numerous island-like or protruding structures on the NPSi substrate, as shown in Figure 6a. These structures have heights of approximately 2 μm. The presence of such pronounced surface features can be attributed to the inherently porous nature of NPSi, leading to the formation of these protrusions during its fabrication process. Notably, these surface protrusions may enhance local electric fields, thereby promoting improved photon absorption efficiency. However, their roles in the context of PEC, reflectance, and fluorescence require further investigation. The SEM images revealed a remarkable change in surface morphology after Ni deposition, as shown in Figure 6b. Not only does Ni adorn the tops of the existing protrusions but evidence also suggests that Ni infiltrates the lower regions of the NPSi structure. This infiltration represents a multifaceted structural change, with Ni not only augmenting the protrusions but also penetrating deeper layers. This coordinated interaction between NPSi and deposited Ni creates a unique composite architecture that might affect the transport and distribution of charge carriers.

These SEM observations elucidate the many facets of the phenomenon. The preservation of low reflectance, crucial for efficient photon absorption in PEC processes, can be partly associated with the persistence of island-like or protruding structures on the NPSi surface after Ni deposition. These structures, although possibly modified by Ni infiltration, continue to provide enhanced light-trapping effects, thus enabling higher photon absorption rates and improved PEC performance. The unchanging PL characteristics despite Ni deposition may also be explained by the SEM results. The penetration of Ni into the NPSi structure constitutes a profound modification to the surface electronic structure, which could affect charge transport dynamics. This modification in maintaining PL properties may also help to maintain efficient charge separation and reduce recombination, positively affecting both PEC and PL results.

### 3.3. PEC Performance of Ni-Deposited NPSi Using Different Ni-Deposition Conditions

**Figure 7:**
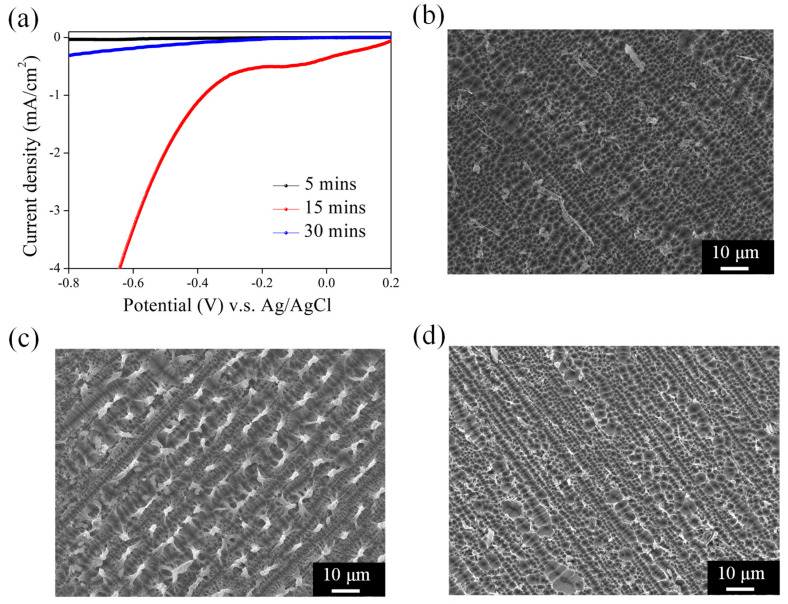
(**a**) PEC J–V curves of Ni-coated NPSi fabricated using different Ni deposition durations. SEM images of Ni-coated NPSi using (**b**) 5, (**c**) 15, and (**d**) 30 min deposition times.

Findings on the relationship between Ni deposition time and the PEC behavior of NPSi provide greater nuance in the optimization of this composite system. This study thus deposited Ni over 5, 15, and 30 min in experiments. The results revealed a pattern wherein the 15-min Ni deposition exhibited the most favorable PEC performance, as shown in Figure 7a. This enhancement in PEC efficiency following a 15-min Ni deposition offers valuable insights into the role of deposition duration in modulating surface characteristics. This behavior is consistent with the intricate relationship between surface morphology, charge carrier dynamics, and catalytic activity. The optimal PEC performance at the 15-min Ni deposition duration might result from a balanced combination of factors. The deposition process in introducing Ni into the NPSi structure requires an optimal timeframe to ensure that the catalytic species is efficiently and uniformly distributed. A shorter deposition duration might not provide adequate coverage, leading to fewer optimal catalytic sites for the PEC reaction [46]. Conversely, an extended deposition time could result in the formation of overgrown Ni clusters, impeding charge carrier transport and hampering the catalytic surface accessibility. Furthermore, the 15-min Ni deposition duration may substantially affect the surface electronic structure of NPSi. The surface modification by Ni infiltration might reduce charge carrier recombination pathways, thereby enhancing their separation efficiency. This may be a factor contributing to the observation of improvement in PEC performance. Findings on the effect of Ni deposition time on the surface morphology of NPSi help us better understand the factors affecting PEC performance. Three distinct Ni deposition durations—5, 15, and 30 min—yielded SEM images, as shown in Figure 7b–d. The 15-min Ni deposition resulted in a notable increase in surface protrusions, unlike the 5-min and 30-min depositions, which lacked such features. The conspicuous presence of surface protrusions on the NPSi structure after a 15-min Ni deposition warrants careful examination. This occurrence might be explained by the coordinated interaction between the deposition duration and the growth kinetics of Ni clusters on the NPSi surface. A moderate deposition time of 15 min appears to encourage the controlled growth of Ni species, leading to the formation of distinct surface protrusions. These protrusions could stem from the preferential deposition of Ni on specific regions of the NPSi surface, which reflects variations in local surface reactivity. This observed surface morphology is closely tied to the PEC performance of the composite system [47]. The existence of surface protrusions, as depicted in the SEM images, potentially enhances light absorption by promoting multiple reflections within the porous structure, thus increasing the optical path length for incident photons. This increased light harvesting could contribute to the improved PEC performance associated with the 15-min Ni deposition. Furthermore, the surface protrusions might also serve as effective charge separation sites. By spatially separating charge carriers and minimizing their recombination, these structures could contribute to the increased photocurrent density observed in the PEC measurements. This behavior corresponds with the well-established understanding that efficient charge separation and transport are crucial determinants of PEC efficiency. Conversely, the absence of noticeable surface protrusions in the SEM images for the 5-min and 30-min Ni depositions indicates a potential limitation in light absorption and charge separation efficiency. The lack of these surface features might hinder light trapping and efficient charge carrier separation, leading to relatively lower PEC performance.

**Figure 8:**
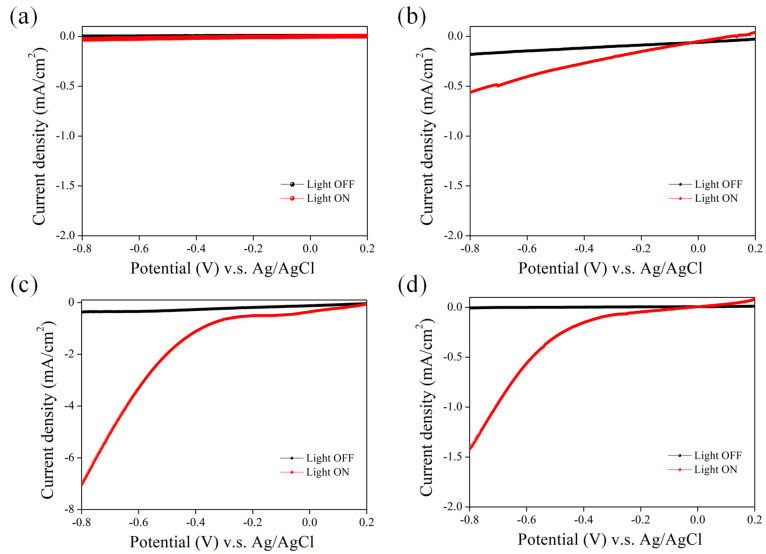
PEC J-V curves of Ni-coated NPSi photocathodes fabricated using (**a**) 40 °C, (**b**) 50 °C, (**c**) 60 °C, and (**d**) 80 °C before (light OFF) and under white illumination (light ON). The Ni deposition duration was fixed at 15 min.

The effect of distinct Ni deposition temperatures on the PEC behavior of the NPSi was also investigated. Our experimental design encompassed four distinct Ni deposition temperatures—40 °C, 50 °C, 60 °C, and 80 °C. The results indicated that the PEC performance was most favorable at a Ni deposition temperature of 60 °C, as shown in Figure 8. The enhancement in PEC performance at this temperature necessitates thorough analysis. This observation can be attributed to the intricate relationship between the deposition temperature and the underlying physicochemical processes governing the Ni deposition process. At a deposition temperature of 60 °C, the kinetics of Ni nucleation, growth, and aggregation could be coordinated in a manner that optimally enhances the properties of the Ni−NPSi hybrid structure. The favorable PEC performance observed at 60 °C may arise from multiple interrelated mechanisms. First, deposition temperature plays a crucial role in determining the crystalline structure and adhesion properties of the deposited Ni species. A deposition temperature of 60 °C might foster the formation of a conformal and adherent Ni layer on the NPSi surface, minimizing charge carrier recombination and enabling efficient charge extraction. Second, the energy distribution of the deposited Ni clusters may be optimal at 60 °C, thereby promoting catalytic activity for the PEC reaction. Ni clusters formed at this temperature might possess the ideal surface electronic structure, which may enhance the water-splitting reaction, leading to an increase in photocurrent density. Moreover, the 60 °C deposition temperature could create a balanced distribution of Ni clusters across the NPSi surface, ensuring uniform catalytic activity and charge separation efficiency. These temperature-induced alterations in surface morphology and electronic structure could collectively contribute to the observed improvement in PEC performance. Contrastingly, deviations from the optimal deposition temperature, observed at 40 °C, 50 °C, and 80 °C, could lead to variations in Ni cluster size, distribution, and adhesion. Such factors might hinder the desired catalytic activity and charge separation efficiency, resulting in less favorable PEC performance.

### 3.4. Long-Term Stability of PEC Performance of Ni-Deposited NPSi

**Figure 9:**
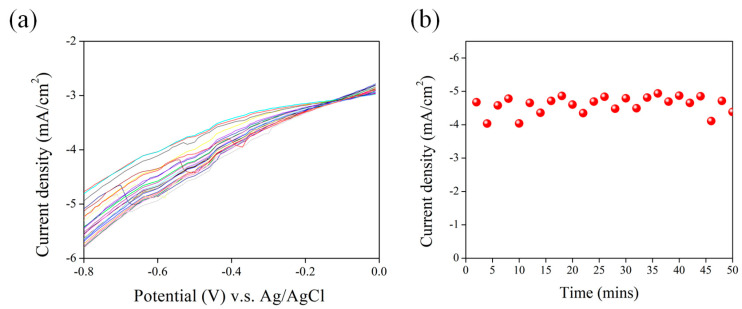
(**a**) PEC J-V curves of Ni-coated NPSi photocathodes fabricated using 60 °C temperature and 15 min deposition time. Each J-V curve was recorded every 2 min. (**b**) The 50-min current density measurement for Ni-coated NPSi photocathodes at bias potential of −0.6 V.

The examination of the long-term stability and transient response of the Ni−NPSi hybrid system to continuous white light exposure, combined with the periodic application of voltage at −0.6 V, has revealed a notable observation. During the 50-min illumination period, the PEC performance demonstrated remarkable stability, where it maintained consistent fluctuation between −4 and −5 mA/cm^2^, as illustrated in Figure 9. This phenomenon warrants thorough analysis. The observed steady-state behavior can be attributed to an intricate interplay between the intrinsic properties of the Ni−NPSi hybrid system, the light irradiation, and the applied voltage. Under continuous white light illumination, the NPSi component of the hybrid system undergoes photon-induced electron-hole pair generation, contributing to steady photocurrent generation. Simultaneously, the deposited Ni species catalyze the water-splitting reaction, producing a continuous supply of hydrogen and oxygen. The observed stability in the photocurrent density, ranging between −4 and −5 mA/cm^2^, indicates a balanced dynamic equilibrium between photon-induced charge generation and the catalytic reactions occurring at the Ni−NPSi interface. This equilibrium is maintained through the synergy between efficient charge separation and catalytic activity, which stems from the unique properties of the Ni−NPSi hybrid structure. The Ni catalyst aids in the charge transfer processes, preventing excessive charge recombination and the NPSi substrate provides a steady source of charge carriers. Furthermore, the applied voltage of −0.6 V assists in the directional movement of charge carriers towards the electrode, aiding in the maintenance of a stable photocurrent. This voltage-induced drift counterbalances the natural tendency of charge carriers to recombine, leading to the observed stability in PEC performance.

## 4. Conclusions

In this study, we have extensively explored the photoelectrochemical (PEC) behavior of nanoporous silicon (NPSi) and Ni-coated NPSi in a hybrid system. Our investigations have shed light on the intricate factors influencing PEC performance, leading to several key findings. Initially, we characterized the PEC performance of uncoated NPSi and observed limitations in photocurrent density under a positive bias. Subsequently, by coating NPSi surfaces with Ni, we achieved a remarkable enhancement in PEC performance, resulting in a substantial increase of up to −22.5 mA/cm^2^ in maximum photocurrent density. This enhancement can be attributed to the catalytic activity enabled by the Ni coating, which facilitates water dissociation and augments the light absorption capability of NPSi. Additionally, the Ni coating modulates carrier transport and recombination processes. Furthermore, our scanning electron microscopy (SEM) analysis revealed distinctive protruding structures on the NPSi surface, which became Ni-covered after coating, extending to the base of the NPSi. This structural transformation correlates with enhanced PEC performance, as these protrusions support the generation of electron-hole pairs, intensify carrier mobility and distribution, and consequently enhance photocurrent density. We also explored the effects of varying Ni deposition times and temperatures on PEC performance. Notably, a 15-min Ni coating time demonstrated optimal PEC performance, likely due to the formation of suitable protruding structures within a moderate timeframe. This enhances the surface electric field and encourages carrier motion without causing excessive Ni deposition. Furthermore, our study highlighted that PEC performance is most favorable at a Ni deposition temperature of 60 °C, owing to the uniform coverage of NPSi surfaces by Ni at this temperature, which preserves the desired catalytic activity and surface electronic structure. Finally, we subjected the Ni−NPSi hybrid system to continuous 50-min white light illumination, resulting in stable fluctuations between −4 and −5 mA/cm^2^ in PEC performance under a bias potential of −0.6 V. This behavior arises from the synergistic coupling of the PEC characteristics of NPSi and the catalytic efficiency induced by Ni coating, combined with the influence of an applied external bias. This interaction ensures continuous carrier separation and transport. Our findings offer valuable insights into the design and optimization of light-driven water-splitting catalysts. By elucidating the critical role played by Ni-coated NPSi in enhancing PEC performance, we contribute to the broader field of renewable energy conversion. These insights pave the way for the development of more efficient and sustainable energy conversion technologies, aligning with the global pursuit of clean and renewable energy sources.

## Data Availability

Data is unavailable due to privacy.
